# Beauvericin potentiates the activity of pesticides by neutralizing the ATP-binding cassette transporters in arthropods

**DOI:** 10.1038/s41598-021-89622-5

**Published:** 2021-05-25

**Authors:** Charbel Al Khoury, Nabil Nemer, Georges Nemer

**Affiliations:** 1 Department of Natural Sciences, School of Arts and Sciences, Lebanese American University, Byblos Campus, P.O. Box 36, Byblos, Lebanon; 2 Department of Agriculture and Food Engineering, Holy Spirit University of Kaslik, P.O. Box 446, Jounieh, Lebanon; 3 Department of Biochemistry and Molecular Genetics, Faculty of Medicine, American University of Beirut, P.O. Box 110236, Beirut, Lebanon; 4 Division of Genomics and Translational Biomedicine, College of Health and Life Sciences, Hamad Bin Khalifa University, P.O. Box 34110, Doha, Qatar

**Keywords:** Transcriptomics, Environmental sciences

## Abstract

Multi-drug resistance is posing major challenges in suppressing the population of pests. Many herbivores develop resistance, causing a prolonged survival after exposure to a previously effective pesticide. Consequently, resistant pests reduce the yield of agricultural production, causing significant economic losses and reducing food security. Therefore, overpowering resistance acquisition of crop pests is a must. The ATP binding cassette transporters (ABC transporters) are considered as the main participants to the pesticide efflux and their neutralization will greatly contribute to potentiate failed treatments. Real-Time PCR analysis of 19 ABC transporter genes belonging to the ABCB, ABCC, ABCG, and ABCH revealed that a broad range of efflux pumps is activated in response to the exposure to pesticides. In this study, we used beauvericin (BEA), a known ABC transporters modulator, to resensitize different strains of *Tetranychus urticae* after artificial selection for resistance to cyflumetofen, bifenazate, and abamectin. Our results showed that the combinatorial treatment of pesticide (manufacturer’s recommended doses) + BEA (sublethal doses: 0.15 mg/L) significantly suppressed the resistant populations of *T. urticae* when compared to single-drug treatments. Moreover, after selective pressure for 40 generations, the LC_50_ values were significantly reduced from 36.5, 44.7, and 94.5 (pesticide) to 8.3, 12.5, and 23.4 (pesticide + BEA) for cyflumetofen, bifenazate, and abamectin, respectively. While the downstream targets for BEA are still elusive, we demonstrated hereby that it synergizes with sub-lethal doses of different pesticides and increases their effect by inhibiting ABC transporters. This is the first report to document such combinatorial activity of BEA against higher invertebrates paving the way for its usage in treating refractory cases of resistance to pesticides. Moreover, we demonstrated, for the first time, using in silico techniques, the higher affinity of BEA to ABC transformers subfamilies when compared to xenobiotics; thus, elucidating the pathway of the mycotoxin.

## Introduction

Insecticide resistance is a major nuisance throughout the world, with approximately 500 different insects (crop pest, parasites of livestock, common urban pests, and disease vectors) species having become less susceptible to about 400 molecules^1^. In insecticide resistance management, the main challenge is to uncover the natural etiology that caused a given population to evolve towards a resistant phenotype and its consequences on the genetic modification of the arthropod. Therefore, it is crucial to intercede in the resistance acquisition of the insect and identify means of suppressing its damaging effect. Insecticide resistance usually involves one or more of the following mechanisms: behavioral changes, mutations leading to changes in the sensitivity of the target site, and overexpression of major detoxification genes including ATP-binding cassette transporter (ABC transporters) esterases, glutathione-S-transferases (GST), and the cytochrome P450 superfamily^2–6^.

Alyokhin et al.^7^ reasonably described the situation of commercial agriculture by being stuck on the “insecticide treadmill”: (i) a novel molecule is newly introduced to the market, (ii) target pests acquire resistance, (iii) an alternative molecule is presented and utilized until it becomes unsuccessful. This approach has proven successful in the past; however, it is facing major limitations in the long run. Nowadays, significantly fewer molecules are produced in the market due to increasingly higher production costs, in addition to their use and application restrictions. Moreover, some of the previously introduced molecules are being banned or their use severely restricted by multiple countries. All reasons considered, conserving the efficacy of available insecticides has become crucial for those involved in commercial agriculture^8^.

ATP Binding Cassette (ABC) proteins are found in all free-living organisms^9^. These proteins participate in the efflux of numerous compounds, including but not limited to, ions, amino acids, lipids, lipopolysaccharides, peptides, chemotherapeutics drugs, allelochemicals, and xenobiotics^10^. Structurally, a functional transporter contains 2 nucleotide-binding domains (NBD) and 2 transmembrane domains (TMD). The NBD is a highly conservative domain that provides energy from ATP hydrolysis to transport different substrates across the membrane. The TMD, consisting of six membrane-spanning helices, offers specificity for the substrates^11^. Arthropods contain several genes encoding for ABC transporters that are thought to play a key role in the emergence and dynamics of pesticide resistance^12–14^. The involvement of these proteins in the pesticide resistance acquisition has been previously demonstrated in many pests such as *Aedes aegypti*^15,16^, *Tribolium castaneum*^17^, *Helicoverpa armigera*^18,19^*,* and *Drosophila melanogaster*^20,21^*.*

Beauvericin (BEA), a cyclic hexadepsipeptide , is the most famous toxin produced by the entomopathogenic fungus *Beauveria bassiana* (Balsamo) Vuillemin (Hypocreales: Cordyciptaceae). A growing array of consolidated data shows that BEA has diverse bioactivities and is being considered a potential candidate for medicinal research. The antibacterial activity of BEA has been investigated against gram-negative and positive bacteria of human, animal, and plant interest^22–24^. A study conducted by Zhang et al.^25^ demonstrated the activity of BEA against the yeast *Candida albicans.* The authors proved that mycotoxin could synergize the effect of a low dosage of ketoconazole and could be used in combination to develop new anti-fungal therapeutics. Zhang et al.^26^ showed the cytotoxic activity against the cancer cell lines PC-3M (prostate) and MDA-MB-231 (breast). Additionally, Heilos et al.^27^ further investigated the anti-cancer properties of BEA where the mycotoxin was able to significantly reduce CT-26 and KB-3-1-grafted tumors in a murine model. Recently, results obtained by Al Khoury et al.^28^ revealed high efficacy of the molecule against all developmental stages of the mite *Sarcoptes scabiei* and might be considered for the treatment of sarcoptic infections. A study conducted by Shin et al.^29^ demonstrated the inhibitory effect of the mycotoxin against the purified retroviral enzymes involved in the integration of the human immunodeficiency virus type 1 (HIV-1). The mechanism of action of BEA is not known yet. Kouri et al.^30^ suggested that BEA can increase ion permeability in biological membranes due to its ionophoric characteristics. Moreover, Jow et al.^31^ indicated that BEA can induce cell death by decreasing the mitochondrial transmembrane potential, releasing cytochrome c, and activating caspase-3. Recently, several studies revealed that BEA can interact with ABC transporters influencing the bioavailability, pharmaco(toxico)kinetics, and efficacy of xenobiotics and pharmaceuticals^32,33^. Drug efflux is the most important determinant of multi-drug resistance, the modulator of the efflux mechanism may be considered as an effective solution to overpower resistance. In this study, we aim to investigate the ability of BEA to reverse the resistance acquisition of *Tetranychus urticae* Koch (Trombidiformes: Tetranychidae) to the most commercially used pesticides by blocking ABC transporters. Moreover, the transcription profile of ABC transporters involved in the efflux of drugs was inspected. Finally, we explored the binding of BEA against different subfamilies of ABC transporter using a molecular docking approach.

## Materials and methods

### Molecules to be evaluated

Three commercial acaricides cyflumetofen (Danisaraba, OAT Agrio Co., Ltd), bifenazate (Floramite, Arysta), and abamectin (Tervigo, Syngenta) were tested alone and in combination with BEA (Sigma-Aldrich). All molecules were stored according to the manufacturer’s recommendations.

### *Tetranychus* mite

The two-spotted spider mite, *Tetranychus urticae* Koch has been used as a model organism throughout this study because its resistance to a large number of compounds with a different chemical structure and mode of action has been previously documented^34,35^. The laboratory susceptible strain of *T. urticae* (LS-TSM) was originally collected from *Phaseolus vulgaris* L. and maintained under laboratory conditions (temperature 23–25 °C and a photoperiod of 16:8 h) with no exposure to any pesticide. To obtain pesticide-resistant strains, *P. vulgaris* leaf disc containing 100 motile stages (nymphs and larvae) of LS-TSM strain were exposed to successive doses of the pesticides (cyflumetofen, bifenazate, or abamectin) to exert selection stress that killed about 50–70% of the initial population of mites. At 72 h post-spraying, mites that survived were transferred to unsprayed *P. vulgaris* plants and left 48 h for oviposition, then all motile stage were removed leaving newly laid eggs only. After egg hatching, emerged motile stages were transferred back to a new *P. vulgaris* leaf disc and sprayed once again with the pesticide. At each generation, the concentration of the pesticide was adjusted to exert selection stress that killed 50–70% of the population. After every 5 generations, the efficacy of the different pesticides was evaluated against *T. urticae* and the lethal concentration necessary to kill half of the mite’s population (LC_50_) was calculated for the generation. After 40 generations of artificial selection, the resistant strains to cyflumetofen, bifenazate, and abamectin were called CR-TSM, BR-TSM, and AR-TSM, respectively. Resistance ratios (RRs) were calculated by dividing the LC_50_ value of the strain under investigation by the LC_50_ value of the LS-TSM strain. This experiment was repeated 3 times in triplicates.

### Ability of BEA to reverse resistance to pesticides

The possibility of BEA to potentiate the activity of cyflumetofen, bifenazate, and abamectin was assessed on the artificially selected resistant strains CR-TSM, BR-TSM, and AR-TSM respectively. Assays were performed by spraying combination doses of the cyflumetofen, bifenazate, or abamectin mixed with BEA (0.15 mg/L). A pilot study was conducted to select a convenient dose of BEA that would not be held accountable per se for the mortality of the mites. For that, a dose–response experiment consisted of numerous concentrations of BEA sprayed on the wild strain of the mite, the selected concentration of 0.15 mg/L was the highest dose that did not record a significant difference when compared to the control (water). LC_50_ values of the pesticide against the resistant strains (CR-TSM, BR-TSM, and AR-TSM) were calculated, and mites sprayed with a mixed dose of pesticide and BEA were considered as a test while mites that received a single pesticide dose were considered as control.

### *T. urticae* resistance acquisition to combination drugs

Selection by combinational drug (pesticide + BEA) resistance was performed as described above (“*Tetranychus* mite” section) using LS-TSM as a parental strain. At each generation, the concentration of the pesticide was adjusted to exert selection stress that killed 50–70% of the population; however, the concentration of BEA remained at 0.15 mg/L during the experiment. After every 5 generations, the efficacy of the drug combination was evaluated and LC_50_ was calculated. After 40 generations of artificial selection by combination drugs (pesticide + BEA), the RRs of the mites were compared to those obtained from the selection by single drugs (pesticide) (“*Tetranychus* mite” section). This experiment was repeated 3 times in triplicates.

### Expression analysis of genes encoding for ABC transporters

After every 5 generations of artificial selection, RNA was extracted from ~ 500 mites (nymphs and adults) using Zymo Research kit according to the manufacturer's protocol. The differential transcriptomics assessed the potential involvement of 19 genes encoding for ABC transporters in the ABCB (2 genes), ABCC (4 genes), ABCG (4 genes), and ABCH (9 genes) subfamilies owing to their previously exhibited inclusion in the detoxification reactions of xenobiotics (pesticides and allelochemicals). The values collected from the wild and resistant strains of *T. urticae* were considered as calibrator and test values, respectively. The mean of Ct values gathered from mites reared on plants sprayed with water + 0.1% Tween 80 were considered as control. A list of the gene accession numbers, primer sequences, amplification efficacies, and amplicon size are presented in Table 1. The concentration of the RNA was estimated by Nanodrop and its integrity was assessed by agarose gel (1%) electrophoresis. Two µg total RNA were transcribed using iScript (Biorad, USA) according to the manufacturer’s protocol. The reaction and cyclic conditions were performed as described by Al Khoury et al.^36^. Actin was used as a reference gene for *T. urticae* due to its demonstrated expression stability^37^. The PCR efficiency was estimated by standard curves designed from Ct values plotted versus the log of concentrations (tenfold serial dilutions) and calculated as E = 10^[−1/slope]^. The amplification specificity was shown by the single melting peak in addition to the amplicon size on gel electrophoresis. Negative controls (no template) were run for each gene to detect primer dimerization and unspecific amplification. The qPCR analysis was repeated 3 times in triplicates, and the mean values were calculated for the gene expression analysis. The relative differences in expression were calculated using the 2^−ΔΔCq^^38^.

**Table 1:** Arthropod primers used in this study.

Gene ID	Subfamily	Sequence	Amplicon size	Amplification efficacy	References
tetur11g04030	B	TTGGCACGAGATGCTCCAAT	151	97.6	This study
		GAAGAGGGACCATCGCAGTT			
tetur11g04040	B	TCAGGATCGTTTGCTCGGAC	123	101.2	This study
		GAGCAGGAGCGTGAGTTTCT			
tetur09g04620	C	ACCGGCTCCGTCTGATTTTT	141	99.7	This study
		CCGACTGTTTGGAGGAGCAT			
tetur01g10390	C	CCAAGACCTGAATGGCCAGA	146	101.2	This study
		GCACCAGTTCGACCCACTAT			
tetur03g09800	C	TTGGTATTGTCGGGTCTGGC	147	102.6	This study
		TGTTGTCTCGGACTGTTGCAT			
tetur28g01950	C	GGCCGTTGATATCCAGCGTA	116	97.9	This study
		ACGAAACACCCAATTGTCGC			
tetur04g04550	G	GGCGGTCAAAAGAAGCGAAC	88	100.4	This study
		CCAGACCCGATGTTGGTTCA			
tetur09g01930	G	TGTGGCATTGGCTTACTGGG	78	99.7	This study
		ATCGAAAGGAAAATAGCTCCGT			
tetur37g01090	G	CTTTGCCTGGGTCGTTGTTG	130	100.9	This study
		GGCCTGTTGTAGGTTCGTCA			
tetur02g11400	G	TATGGGTCCAAGTGGTGCTG	131	100.7	This study
		CATGTTGCGGAATGAACGCA			
tetur12g03910	H	ATCGGCAATCCTCCAACAGG	160	103.3	This study
		GTGCTTTACGTGCATCAGCC			
tetur18g00230	H	AAAAGTGCCGAAAGGAGCCA	137	101.4	This study
		TCTCTAACGGGGAACGACCA			
tetur21g00940	H	ATGCCAGCGTTTACCCAACT	106	101.8	This study
		CAATCCGGCTGCTTTGAGAC			
tetur03g05300	H	CGGGTGTGGTAAAACTTCGC	84	102.5	This study
		TTAGAGCCGAAAACCCGGAC			
tetur01g03530	H	TGGATTTGCCCTTACCGCAT	197	98.6	This study
		ATGACCCACGACAGGGGATA			
tetur01g05940	H	TGCTTCGATGTGTTGTTGGTC	70	98.1	This study
		CCAGGGCGAAAACCAAATATCC			
tetur13g02010	H	TTGGCTCCATTTTCTGGCCT	165	102.9	This study
		GTGCCCAAGCATACAAGACAC			
tetur04g06390	H	AGTGCCAAAGAGAAGGCGAT	140	100.2	This study
		GCAATGACGGTGGGACATCT			
tetur13g02060	H	GCCACAAGAGGTTGCCCTAA	135	99.3	This study
		TCGTTGATTGGAGGCTCTGC			
tetur03g09480	Actin	GCCATCCTTCGTTTGGATTTGGCT	113	99.7	Dermauw et al. 201
		TCTCGGACAATTTCTCGCTCAGCA			

### Homology modeling of *T. urticae* ABC transporters and docking of BEA

The ABC transporters belonging to the ABCB (2 genes) and ABCC (4 genes) subfamilies were selected for molecular docking. The structures of *T. urticae* ABC transporters were modeled using Alignment Mode algorithm of SWISS-MODEL^39–41^ using the mouse P-glycoprotein (PDB ID: 4M1M) and the bovine multidrug resistance protein 1 (PDB ID: 6UY0) and as templates (Table 2). We selected the templates similar to the query sequence coverage and identity of the sequence-based upon their GlobalModel Quality Estimate (GMQE). A 3D-refine software was used for the refinement of *T. urticae* ABC transporters modeled structures by optimizing the hydrogen bonds as well as the atomic-level energy^42^. The best-refined model was selected based on the MolProbity score; the lower score shows the excellent model^43^ (Table 2).

**Table 2:** Details of scores obtained for target ABC transporters by Swiss-model and 3D-refine servers.

Gene ID	Template used for homology modeling	Sequence identity (%)	Ramachandran score (% of residues)	GMQE score	Mol probity	Ramachandran score after refinement (% of residue)
tetur11g04030	4M1M	45.02	93.91	0.73	1.12	94.66
tetur11g04040	4M1M	40.88	94.41	0.65	1.4	95.34
tetur09g04620	6UY0	47.22	93.88	0.65	1.06	95.85
tetur01g10390	6UY0	42.31	93.59	0.63	0.96	95.88
tetur03g09800	6UY0	44.19	94.32	0.65	1.39	95.27
tetur28g01950	6UY0	45.77	95.62	0.81	0.82	96.87

For the molecular docking, the crystal structure of BEA was downloaded in sdf format (2d format) from The Toxin and Toxin Target Database (www.t3db.ca/toxins) and transformed into a 3D structure automatically during the docking process. The refined homology structures of the ABC transporters were used as receptors. The PDB format of the proteins were loaded into Molsoft.icm-pro v3.9-1b^44^ and converted into an ICM object (selected options: optimize hydrogen, optimize HisProAsnGlnCys, hide missing side chains). Three drugs with potential inhibition activities against ABC transporters were used as positive control (i) cyclic-tris-(S)-valineselenazole “QZ59RRR” (PubChem ID: 25195366), (ii) cyclic-tris-(R)-valineselenazole QZ59RRR (PubChem ID: 25195367), stereoisomeric analogs of dendroamide-A with demonstrated activities against mouse P-glycoprotein^45^ and (iii) the multidrug resistance reverter verapamil (PubChem ID: 2520)^46^. The binding affinity of BEA was compared to cyflumetofen (PubChem ID: 11496052), bifenazate (PubChem ID: 176879), and abamectin (PubChem ID: 6435890). The energy map was set upon the substrate binding site (6000 Å^3^) of the ABC transporters^47^ where the hydrogen bonding potential, van der Waals potential with carbon-, sulphur- and hydrogen-like probes, hydrophobic potential, and electrostatic potential are taken into consideration. The conformational examination in the program depends on the Biased probability Monte Carlo (BPMC) system^48^. Docking were performed with static target crystal structures and repeated 3 times. The thoroughness was increased to 10 and the best conformations (lowest ICM scores) of BEA were calculated.

### Statistical analysis

LC_50_ values and their standard error were calculated from probit regression using the Statistics for Windows, version 25.0 (SPSS). The relative expression data were analyzed by the statistical comparison test of means (one-way ANOVA). The efficacy of the drugs (pesticide and pesticide + BEA) against *T. urticae* was analyzed by Kaplan Meier survival curves using SPSS (version 25) software. The statistical differences between data obtained with each treatment and the control for each experiment were measured by the Log-rank test expressed by Chi-2 results and P-values using SPSS. The Tukey test for the separation of means, at the 5% threshold, was used.

## Results

### Artificial selection of *T. urticae* resistant strains

The toxicities of cyflumetofen, bifenazate, and abamectin to the different generations of *T. urticae* are shown in Table 3. Prior to pesticide selection, the LC_50_ values of the susceptible strain (LS-TSM) were 0.67, 0.69, and 0.78 mg/L using cyflumetofen, bifenazate, and abamectin, respectively (Table 3). The artificial selection of the LS-TSM strain resulted in a slow resistance acquisition to cyflumetofen and bifenazate relative to the fast increase in the resistance to abamectin (Table 3). After 40 generations, the LC_50_ for cyflumetofen, bifenazate, and abamectin for the resistant strains were 36.5 (CR-TSM), 44.79 (BR-TSM), and 94.56 mg/L (AR-TSM), respectively, which is high above the manufacturer’s recommended rates. The RR varied between 54.4- and 121.2-fold indicating strong resistance acquisition of the mite to the different pesticides used in this study.

**Table 3:** Concentration probit mortality data of cyflumetofen, bifenazate, and abamectin (alone or in combination with beauvericin) against *T. urticae* with calculated resistance ratio.

Number of generation selected	Cyflumetofen				Bifenazate				Abamectin			
	Pesticide (mg/L) ± S.E	RR* ± S.E	Pesticide and beauvericin (mg/L) ± S.E.	RR ± S.E	Pesticide (mg/L) ± S.E	RR ± S.E	Pesticide and beauvericin (mg/L) ± S.E.	RR ± S.E	Pesticide (mg/L) ± S.E	RR ± S.E	Pesticide and beauvericin (mg/L) ± S.E.	RR ± S.E
1	0.67 ± 0	1.00 ± 0	0.67 ± 0	1.00 ± 0	0.69 ± 0	1.00 ± 0	0.70 ± 0	1.00 ± 0	0.78 ± 0	1.00 ± 0	0.76 ± 0	1.00 ± 0
5	1.20 ± 0	1.79 ± 0	0.82 ± 0	1.22 ± 0	1.23 ± 0	1.78 ± 0	0.92 ± 0	1.31 ± 0	1.98 ± 0.1	2.53 ± 0.1	1.24 ± 0	1.63 ± 0
10	2.65 ± 0.1	3.95 ± 0	1.33 ± 0.1	1.98 ± 0	2.70 ± 0	3.91 ± 0.2	1.53 ± 0	2.18 ± 0.1	3.94 ± 0.1	5.05 ± 0.2	1.93 ± 0.1	2.53 ± 0.1
15	5.55 ± 0.2	8.28 ± 0.1	1.84 ± 0.1	2.74 ± 0.1	5.82 ± 0.1	8.43 ± 0.2	2.13 ± 0	3.04 ± 0.2	7.98 ± 0.2	10.23 ± 0.4	2.82 ± 0.1	3.71 ± 0.3
20	7.13 ± 0.3	10.64 ± 0.3	2.55 ± 0.2	3.80 ± 0.1	7.78 ± 0.2	11.27 ± 0.3	2.77 ± 0.2	3.95 ± 0.2	15.44 ± 0.5	19.79 ± 0.9	4.09 ± 0.2	5.38 ± 0.3
25	12.30 ± 0.5	18.35 ± 0.6	3.37 ± 0.2	5.03 ± 0.2	14.33 ± 0.3	20.76 ± 0.5	3.62 ± 0.2	5.17 ± 0.2	22.19 ± 0.9	28.44 ± 1.2	5.67 ± 0.3	7.46 ± 0.5
30	16.30 ± 0.8	24.32 ± 0.7	4.20 ± 0.3	6.26 ± 0.2	19.53 ± 0.3	28.30 ± 0.6	5.19 ± 0.2	7.41 ± 0.3	34.16 ± 1	43.79 ± 1.8	9.09 ± 0.4	11.96 ± 0.7
35	21.78 ± 1.1	32.50 ± 0.9	5.61 ± 0.3	8.37 ± 0.3	32.60 ± 0.5	47.24 ± 0.8	7.30 ± 0.3	10.42 ± 0.4	59.50 ± 1.5	76.28 ± 2.3	16.20 ± 0.7	21.31 ± 1.7
40	36.50 ± 1.1	54.47 ± 1	8.39 ± 0.5	12.52 ± 0.4	44.79 ± 0.8	64.91 ± 1.2	12.54 ± 0.3	17.91 ± 0.7	94.56 ± 2.6	121.23 ± 3.5	23.40 ± 1.3	30.78 ± 2.3

*RRs were determined by dividing the LC50 value of TSM-LB_x_ (x being the number of generation) by the LC50 value of TSM-LB_1_ (LR-TSM).

### Ability of BEA to reverse resistance to pesticides

**Figure 1:**
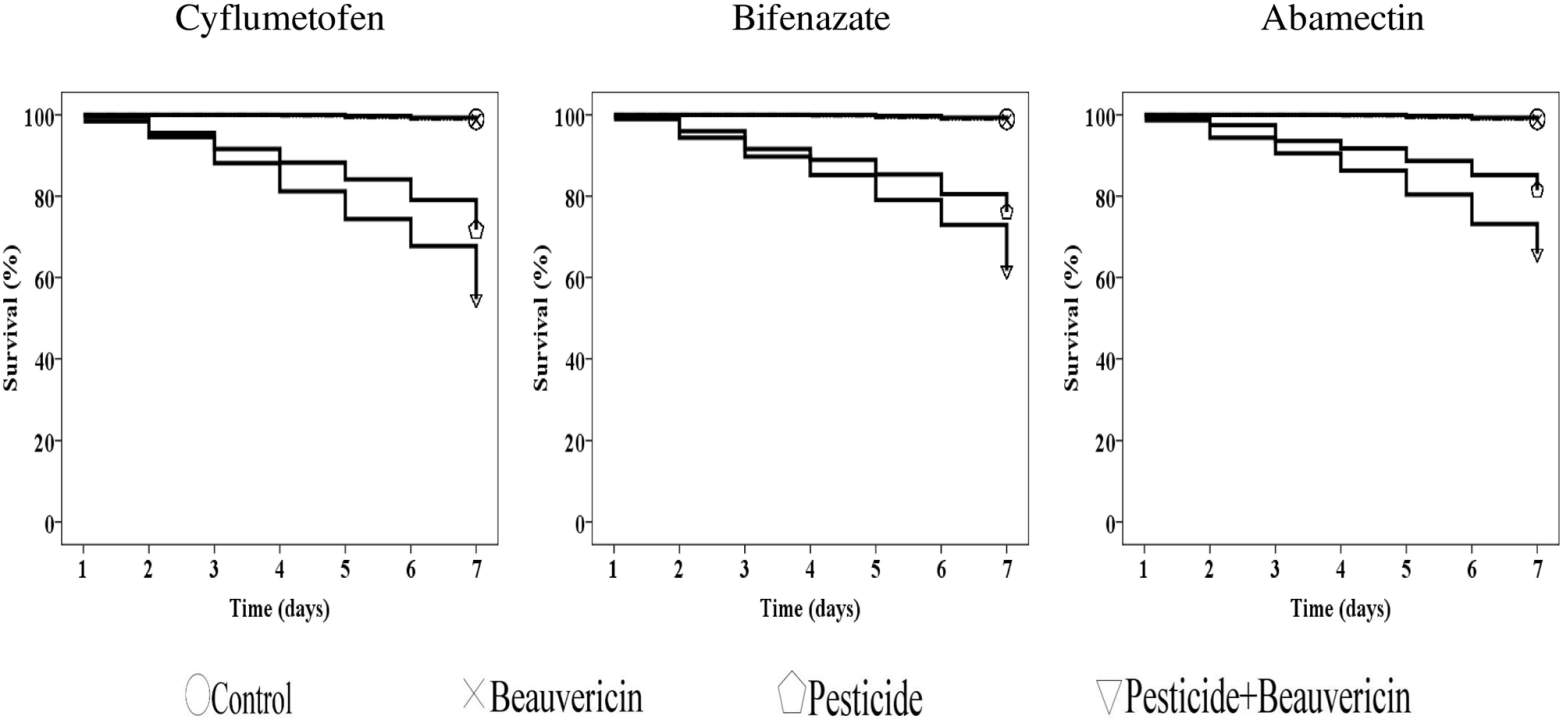
Curves representing survival of *T. urticae* exposed to pesticide molecules (cyflumetofen, bifenazate, or abamectin) alone and in combination with beauvericin.

The efficacy of pesticides, alone or in combination with BEA, was daily observed against motile stages of *T. urticae* for 7 days post-inoculation (DPI). In the pilot study, no significant difference was notable between the survival of mites sprayed with water and those sprayed with BEA (χ^2^ = 0.227; df = 1;* P* > 0.0001) (Fig. 1). After combination with BEA, the efficacy of all drugs has been significantly enhanced relative to the control (pesticide alone) (Fig. 1). Significantly lower survival rates were recorded against motile stages of *T. urticae* exposed to mixed molecules (pesticide + BEA): cyflumetofen + BEA (χ^2^ = 44.897; df = 1;* P* < 0.0001), bifenazate + BEA (χ^2^ = 32.848; df = 1;* P* < 0.0001), and abamectin + BEA (χ^2^ = 45.876; df = 1;* P* < 0.0001) (Fig. 1).

### Synergetic pesticidal effect of BEA

To elucidate its pesticidal effect, the ability of BEA to inhibit resistance acquisition was evaluated. The selection of *T. urticae* resistant strains was conducted as previously described in “*Tetranychus* mite” section. with a slight modification. The low concentration of BEA mixed pesticide dosage significantly counteracted the resistance acquisition (Table 3, Supplementary Table S1). The LC_50_ value against LS-TSM strain were 0.67, 0.7, and 0.76 mg/L using cyflumetofen-, bifenazate-, and abamectin-, respectively, when combined with BEA. As the selection process continued, the RRs of all pesticides in combination with BEA were slowly developing when compared to single doses of pesticides (Table 3, Supplementary Table S1). After 40 generations, the RRs of *T. urticae* selected for resistance to cyflumetofen, bifenazate, and abamectin were 12.52, 17.91, and 30.787. These values were significantly lower relatively to those obtained after the exposure to single doses of cyflumetofen (df = 1, F = 1,814,000, *P *  < 0.05), bifenazate (df = 1, F = 33,140,000, *P * < 0.05), and abamectin (df = 1, F = 102,400, *P * < 0.05) (Table 3, Supplementary Table S1).

### Regulation of ABC transporters

**Figure 2:**
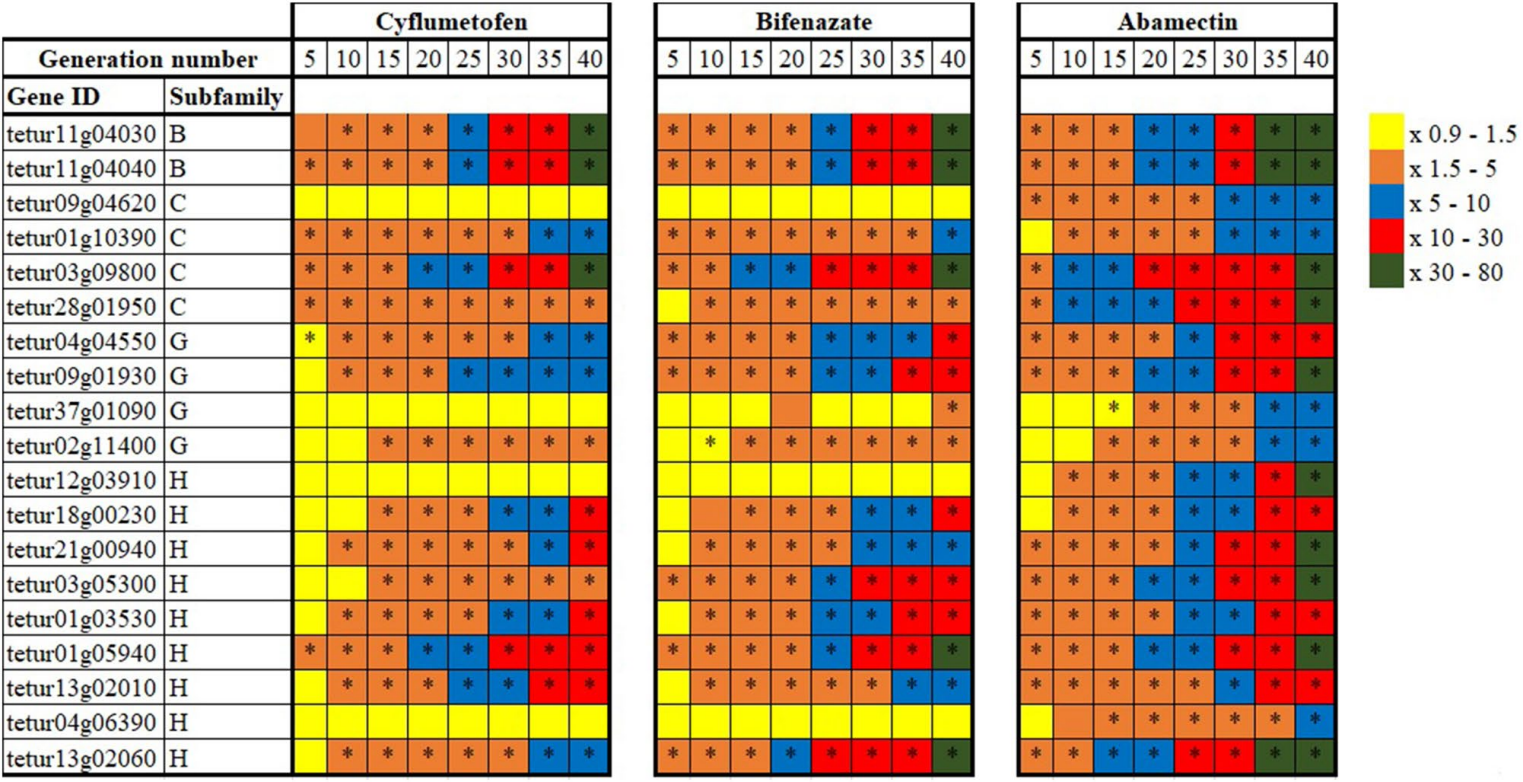
Transcription profiles of 19 ABC transporter genes from the ABCB, ABCC, ABCG, and ABCH subfamilies after the selection for resistance to cyflymetofen, bifenazatem and abamectin. Transcription levels are shown as mean-fold transcription of *T. urticae* resistant strains relative to the laboratory susceptible strain. Asterisks indicate significant differences (one-way ANOVA model, P ≤ 0.05).

The expression levels of 19 ABC transporter genes in different generations of *T. urticae* selected for resistance to cyflumetofen, bifenazate, and abamectin were analyzed. Most of the ABC transporter genes analyzed showed constitutive higher transcription in all generations as the selection process continued (Supplementary Table S2) (Fig. 2). However, no significant difference was notable in the transcription of 4 (tetur09g04620, tetur37g01090, tetur12g03910, and tetur04g06390) and 3 (tetur09g04620, tetur12g03910, and tetur04g06390) genes of *T. urticae* selected for the resistance to cyflumetofen and bifenazate, respectively. In parallel, 15 genes belonging to different ABC transporters subfamilies (B, C, G, and H) were considered as “generalists” and upregulated by *T. urticae* after the exposure to all pesticides (Fig. 2). Furthermore, tetur11g04030, tetur11g04040, tetur03g09800, members of the subfamilies ABCB, ABCB, and ABCC respectively, showed very high expression (30–80- fold change) in *T. urticae* exposed to all molecules. In this study, it was notable that the ABCB subfamily recorded had the highest average expression across mites selected for resistance to cyflumetofen, bifenazate, and abamectin (Supplementary Table S2) (Fig. 2). Finally, the ABC transporters transcriptome response to pesticides in *T. urticae* can be put into the following order based on the number of upregulated genes and their average expression rate: abamectin > bifenazate > cyflumetofen (Fig. 2; Supplementary Table S1).

### Homology modeling of *T. urticae* ABC transporters and docking of BEA

**Figure 3:**
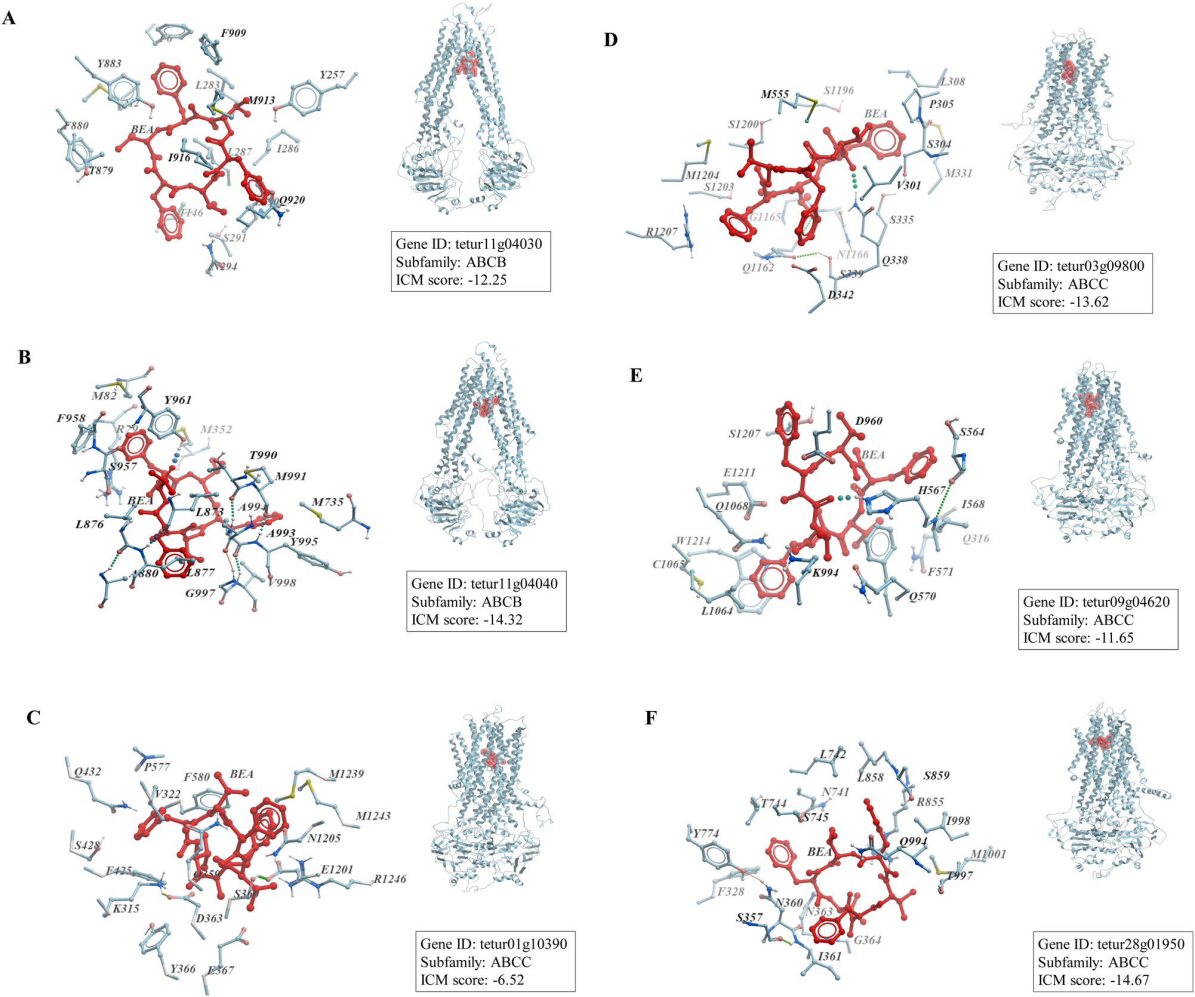
The binding model of BEA against ABC transporters. Interactions between BEA (red) and associated residues (blue) in the interface of the homology models for ABC transporters belonging to the ABC transporter subfamilies B (**A**: tetur11g04030; **B**: tetur11g04040) and C (**C**: tetur01g10390; **D**: tetur03g09800; **E**: tetur09g04620; **F**: tetur28g01950).

Models of ABC transporters belonging to ABCB and ABCC subfamilies were generated using 4M1M and 6UY0 as templates, respectively. The quality of models was estimated using Molprobity score and the structure was validated after the generation of the Ramachandran plot (Table 2) confirming that refined models could be used for molecular docking. Interestingly, BEA recorded the best docking score (Table 4) (Fig. 3) against all ABC transporters (excluding tetur01g10390) when compared to cyflumetofen, bifenazate, and abamectin. In parallel, beauvericin also showed lower affinity relative to the controls QZ59RRR, QZ59SSS, and Verapamil when docked against most full ABC transporters used in this study (Table 4) (Fig. 3). One exception was notable with tetur01g10390, where BEA recorded the highest binding energy (− 6.52 kcal/mol) when compared to QZ59RRR (− 13.1 kcal/mol), QZ59SSS (− 13.23 kcal/mol), and verapamil (− 6.7 kcal/mol) (Table 4) (Fig. 3). The different residue contacts between BEA and the different ABC transporters of *T. urticae* are shown in the Supplementary Table S3).

**Table 4:** ICM score of BEA against ABC transporters implicated in pesticide resistance.

Gene ID	ICM score (kcal/mol)						
	BEA	QZ59RRR	QZ59SSS	Verapamil	Abamectin	Cyflumetofen	Bifenazate
tetur11g04030	− 12.25	− 8.47	− 9.87	− 4.098	5.75	− 10.04	− 9.026
tetur11g04040	− 14.32	− 6.4	− 7.49	− 9.1	− 13.78	− 14.02	− 12.9
tetur09g04620	− 11.65	− 10.51	− 8.6	− 4.16	− 2.4	− 9.7	− 10.53
tetur01g10390	− 6.52	− 13.1	− 13.23	− 6.7	− 8.06	− 8.75	− 2.39
tetur03g09800	− 13.32	− 10.42	− 9.69	− 2.32	− 7.5	− 12.38	− 6.14
tetur28g01950	− 14.67	− 15.45	− 9.12	− 12.75	− 5.95	− 14.45	− 13.79

## Discussion

Historically, pests have always had a pronounced deleterious effect on crop growth, yield, and profit, but now, they pose even a greater threat to the agriculture industry due to their mighty new tool referred to as “multidrug resistance”. The random and excessive utilization of pesticides, in addition to other inadequate agriculture practices, has decreased the susceptibility of the pest population to previously effective molecules. Amid the crises in insect control, scientists have been endeavoring to elucidate the mechanisms behind resistance acquisition^49^. One of the most acknowledged tactics is the expression increasement of efflux pumps to upsurge the elimination of xenobiotics^50^. In addition to other multidrug efflux pumps, ABC transporters are thought to be a key player in translocating pesticide outside the cell membrane^51^. In order to understand their involvement in the development of multidrug resistance, this paper analyzed the expression profile of 19 ABC transporters genes. A study conducted by Labbé et al.^52^ demonstrated that ABCB are linked to pesticide resistance. These observations were further supported by Pohl et al.^53^ who confirmed the participation of ABC genes in the ABCB subfamily in the resistance acquisition of mite *Rhipicephalus microplus.* In addition, the expression profiling revealed that ABC genes in the ABCC, ABCG ABCH subfamilies were differentially expressed in multi-pesticide resistant strains of *T. urticae*^12^. In this study, most of the ABC transporter genes belonging to ABCB, ABCC, ABCG, and ABCH were upregulated after exposure to the different pesticides. It is interesting to note that, after each generation, the higher expression levels of ABC transporters the less sensitive the mite became. Thus, these results support the premise that multiple subfamilies of ABC transporter are implicated in pesticide resistance. Another promising finding was that most of the ABC transporters were modulated by three chemical compounds with different structures and modes of action. A study conducted by Hayashi et al.^54^ discovered that cyflumetofen specifically inhibits mitochondria complex II. Although first thought to be a neurotoxin, Van Nieuwenhuyse et al.^55^ provided genetic evidence that bifenazate targets the mitochondrial complex III. Stumpf et al.^56^ showed that abamectin, a macrocyclic lactone, act as GABA agonists and also bind to glutamate-gated chloride channels in nerve and muscle cells of invertebrates. Despite the fact that each molecule possesses different target sites, we showed that ABC transporters (subfamilies ABCB, ABCC, ABCG, and ABCH) were engaged in multidrug resistance. These results are in accordance with those obtained from Lanning et al.^57^ who demonstrated significantly high expression levels of ABC transporters in the multidrug resistance strain when compared to the susceptible strain of *Heliothis virescens* (Lepidoptera: Noctuidae). Moreover, the expression analysis of *Trichoplusia ni* (Lepidoptera: Noctuidae) ABC transporters validated their potential to contribute to the elimination of pyrethroids^58^. The inclusion of ABC transporters in the multidrug resistance acquisition of *Anopheles arabiensis* was previously shown^59^. Remarkably, One of the ABC genes, that belongs to the ABCG subfamily, is orthologous to the Human HsABCG2 with a validated role in lowering the sensitivity of cancer cells to drugs^60^. The high number of ABC transporters recorded in the *T. urticae* genome is especially attributable to the increase in the numbers of ABCC, ABCG, and ABCH subfamilies. These accelerated subfamilies of ABC transporters were among the most differentially expressed after the transfer of the *T. urticae* from a common into a least favorable host^61^. Similarly, in this study, the members of those ABC subfamilies were highly transcribed by CR-TSM, BR-TSM, and AR-TSM; thus, confirming the dubious reputation of *T. urticae* as the most resistant species^34^. In this study, a very low number of genes lacked significant expression after exposure to cyflumetofen (tetur09g04620, tetur37g01090, tetur12g03910, and tetur04g06390) and bifenazate (tetur09g04620, tetur12g03910, tetur04g06390). Initially, we thought that these genes do not play any role in the efflux of xenobiotics; however, the aforementioned genes were significantly induced after the exposure of *T. urticae* to abamectin. Despite the fact that some of the ABC transporters are implicated in multidrug resistance (generalist activity), it may be hypothesized that some genes are only modulated by a specific drug. These findings are in accordance with those obtained by Dermauw et al.^12^, who showed that numerous ABC transporter genes are expressed under specific environmental conditions. Here we demonstrated, for the first time, that BEA impedes the emergence of resistance and ameliorates the efficacy of pesticides against arthropods. The ability of the mycotoxin to resensitize drug-resistant microorganisms has been previously discussed. In her paper, Szczepaniak et al.^32^ demonstrated that BEA (in addition to another compound in its family, enniantins) can inhibit the activity *Candida albicans* drug efflux pumps; therefore, potentiating the activity of antifungals^26,62^. The most remarkable result to emerge from the data is the capability of BEA to increase the effect of all drugs evaluated in this study. Knowing that multiple ABC transporter subfamilies are implicated in multiple drug resistance, it can be speculated that a minor dose of BEA can synergistically act with different pesticides regardless of the chemical family and mode of action. Another interesting observation is that the combinatorial treatment of pesticides and BEA might come in handy under different circumstances. On one hand, our study demonstrated that BEA could counteract resistance acquisition. After BEA + pesticide treatment, the survival of the *T. urticae* resistant strains (CR-TSM, BR-TRM, and AR-TSM) significantly decreased relative to single pesticide treatment. It may be assumed that this minimal concentration of BEA can target the drug efflux pumps in the resistant strains of *T. urticae*. Consequently, the neutralization of ABC transporters can increase the bioavailability of the pesticide, thus becoming effective again. These results tie well with those from Sharom et al.^63^ who showed that BEA is able to restore the accumulation of chemotherapeutic drugs in multidrug resistant cancer cells. The resistance acquisition of *T. urticae* to 96 different pesticides has been previously documented^64^. At this stage of understanding, the capacity of BEA to reverse pesticide treatment failure and achieve an expected level of control is considered a breakthrough strategy to combat pest resistance to pesticides. On the other hand, our results showed that BEA could initially slow the resistance acquisition of arthropods. After every 5 generations, a significant difference was notable in the RRs of the mite selected with different pesticides + BEA relative to those selected with single doses of pesticides. Despite the notorious ability of the two-spotted spider mite to develop resistance to pesticides, cases of multidrug-resistant field strains are seldomly reported^64^. However, the utilization of BEA would still be recommended due to the high risk of resistance development to many pesticides often after only few applications^34^. The relevance of this study comes from the fact that the concentrations of used drugs for the seletion were similar to those which are used in vivo (according to manufacturer’s instructions). In addition, it was remarkable that LC_50_ and RR values recorded in this study concur well with previous findings^65–68^.Concomitantly, it may be suggested that the ability of BEA to restore pesticide sensitivity could be used under natural conditions and span a wide range of pest species. Furthermore, it is important to mention that the inhibition of the ABC transporters could reduce (but not completely eliminate) drug efflux from the cell. It can thus be reasonably assumed that other enzymes, including, but not limited to, the P450 mono-oxygenases, glutathione-S-transferases, and carboxyl/cholinesterases play a vital role in the metabolic resistance to pesticides. This lends support to findings from Riga et al.^69^ who revealed the overexpression of cytochrome P450 genes is linked with pesticide resistance. In addition, glutathione-S-transferases were associated with lowering the sensitivity of *T. urticae* to Acequinocyl and Pyridaben^70^. Finally, upregulation of carboxyl/cholinesterases (CCEs) has been implicated in several cases of pesticide resistance^71^. The analysis of molecular docking results found the first evidence for BEA inhibition of arthropods ABC transporters. Planned comparisons revealed that BEA is an excellent inhibitor of the drug efflux pumps even when compared to potent molecules^33^*.* Wang et al.^46^ stressed the importance of developing efficient inhibitors to overpower ABC transporter-mediated multidrug resistance. However, one of the major drawbacks to exploiting this strategy is the low potency of these inhibitors^72^. The molecular docking conducted in this study, demonstrated high affinity of BEA to the substrate binding site of the ABC transporter. This correlates satisfactorly with Tong et al.^33^ who showed that BEA is the best drug efflux pump inhibitor of *Candida albicans.* The authors demonstrated the best binding energy for BEA after virtual screening of the 3D ZINC database (≈950 drugs). Taken together, it can be suggested that BEA is the most potent inhibitor for ABC transporters. It is interesting to note that BEA mainly anchors itself to ABC transporters through hydrophobic interactions (Supplementary Table S3). This substantiates previous findings in the literature that the drug binding pocket of ABC transporters is constituted maily of hydrophobic residues^46,47^. Another marked observation to emerge from the data was the difference in the protein residues interacting with BEA when compared to previously demonstrated ABC transporter inhibitors such as doxorubicin and verapamil^46,47^. This may be due to the poly-specific drug binding property of the ABC transporters.In this study, we have showed, for the first time, the most important residues (in arthropods ABC transporters) contributing to the transport of xenobiotics. Further experimental investigations are needed to unveil the possible portals and pathways of BEA to access the binding site in arthropod ABC transporters.

## Conclusion

Altogether, the docking results demonstrate, that BEA binds to the active site of different ABC transporters while having a better binding affinity than pesticides. We hypothesize that BEA might act as a competitive inhibitor of the ABC transporters and inhibit drug transport by preventing substrate association, and consequently, increasing its bioavailability inside the cell. All results considered, we showed that BEA can (i) potentiate “non-efficient” pesticides against resistant strains and (ii) reduce resistance development of susceptible strains. This integrated management can be used as a forceful strategy, with broad therapeutic relevance, in the battle against multidrug resistance.

## Supplementary Information

Supplementary Table S1.

Supplementary Table S2.

Supplementary Table S3.
