# Supplementary material for: Beauvericin potentiates the activity of pesticides by neutralizing the ATP-binding cassette transporters in arthropods
Source: Sci Rep. 2021 May 25;11:10865. doi: 10.1038/s41598-021-89622-5 (PMC8149815; doi:10.1038/s41598-021-89622-5)
Supplement: Supplementary file 1 — Supplementary Table S1. [file 41598_2021_89622_MOESM1_ESM.docx]

**Beauvericin potentiates the activity of pesticides by neutralizing the ATP-binding cassette transporters in arthropods**

**Charbel Al Khoury^1,*^, Nemer Nabil^2^, Nemer Georges^3,4^**

*^1^Department of Natural Sciences, School of Arts and Sciences, Lebanese American University, Byblos Campus, P.O. Box 36, Byblos, Lebanon*

*^2^Department of Agriculture and Food Engineering, Holy Spirit University of Kaslik, P.O. Box 446, Jounieh, Lebanon*

*^3^Department of Biochemistry and Molecular Genetics, Faculty of Medicine, American University of Beirut, P.O. Box 110236 Beirut, Lebanon*

*^4^Division of Genomics and Translational Biomedicine, College of Health and Life Sciences, Hamad Bin Khalifa University, P.O. Box 34110 Doha, Qatar*

*Corresponding author

Phone: +96170543423; E-mail: charbel.alkhoury@lau.edu.lb

Supplementary Table S1: Difference between resistant ratios of *T. urticae* strains selected using pesticides and strains selected using a combination of pesticide & beauvericin. Strains were considered significant when RR of a strain exposed to pesticide alone was different from the RR of a strain exposed to the pesticide mixed with beauvericin after the same number of generations.

| **Generation** | **Cyflumetofen** | | | **Bifenazate** | | | **Abamectin** | | |
| --- | --- | --- | --- | --- | --- | --- | --- | --- | --- |
|  | **df** | **F** | **P** | **df** | **F** | **P** | **df** | **F** | **P** |
| 5 | 1 | 14280 | 0 | 1 | 10220 | 0 | 1 | 36720 | 0 |
| 10 | 1 | 280800 | 0 | 1 | 42280 | 0 | 1 | 38410 | 0 |
| 15 | 1 | 1374000 | 0 | 1 | 650400 | 0 | 1 | 1126000 | 0 |
| 20 | 1 | 2107000 | 0 | 1 | 1200000 | 0 | 1 | 1868000 | 0 |
| 25 | 1 | 548100 | 0 | 1 | 10950000 | 0 | 1 | 2477000 | 0 |
| 30 | 1 | 537100 | 0 | 1 | 560400 | 0 | 1 | 2226000 | 0 |
| 35 | 1 | 774700 | 0 | 1 | 20360000 | 0 | 1 | 73120 | 0 |
| 40 | 1 | 1814000 | 0 | 1 | 33140000 | 0 | 1 | 102400 | 0 |
